# Pinpointing brainstem mechanisms responsible for autonomic dysfunction in Rett syndrome: therapeutic perspectives for 5-HT_1A_ agonists

**DOI:** 10.3389/fphys.2014.00205

**Published:** 2014-05-30

**Authors:** Ana P. Abdala, John M. Bissonnette, Adrian Newman-Tancredi

**Affiliations:** ^1^School of Physiology and Pharmacology, University of BristolBristol, UK; ^2^Department of Obstetrics and Gynecology, Oregon Health and Science UniversityPortland, OR, USA; ^3^Neurolixis Inc.Dana Point, CA, USA

**Keywords:** Rett syndrome, 5-HT_1A_ receptor, breathing, anxiety, motor activity, serotonin, brainstem, vagal tone

## Abstract

Rett syndrome is a neurological disorder caused by loss of function of methyl-CpG-binding protein 2 (MeCP2). Reduced function of this ubiquitous transcriptional regulator has a devastating effect on the central nervous system. One of the most severe and life-threatening presentations of this syndrome is brainstem dysfunction, which results in autonomic disturbances such as breathing deficits, typified by episodes of breathing cessation intercalated with episodes of hyperventilation or irregular breathing. Defects in numerous neurotransmitter systems have been observed in Rett syndrome both in animal models and patients. Here we dedicate special attention to serotonin due to its role in promoting regular breathing, increasing vagal tone, regulating mood, alleviating Parkinsonian-like symptoms and potential for therapeutic translation. A promising new symptomatic strategy currently focuses on regulation of serotonergic function using highly selective serotonin type 1A (5-HT_1A_) “biased agonists.” We address this newly emerging therapy for respiratory brainstem dysfunction and challenges for translation with a holistic perspective of Rett syndrome, considering potential mood and motor effects.

Rett syndrome (RTT) is a neurological disorder caused by loss of function of methyl-CpG-binding protein 2 (MeCP2). This syndrome is most often linked to mutations in the X-linked gene that encodes this protein. The loss of function of this ubiquitous transcriptional regulator has a devastating effect in particular on the central nervous system. The syndrome presents with cortical features, including severe mental disability and epilepsy (Dolce et al., 2013); and extrapyramidal symptoms, resulting in dystonia and dyskinesia (Chahrour and Zoghbi, 2007). However, a life-threatening presentation of this syndrome is the brainstem dysfunction, which results in breathing disturbances. This phenotype, mimicked by the mouse models of RTT, is deemed the most reliable outcome measure for clinical translation (Katz et al., 2012). Like most phenotypes in RTT, the presentation of respiratory disturbances is highly variable, but typically includes episodes of breathing cessation interspersed with hyperventilation or irregular breathing (Ramirez et al., 2013). Defects in various neurotransmitter systems have been observed in patients and animal models of Rett syndrome (Weng et al., 2011), but we believe serotonin (5-HT) deserves particular interest due to its role in promoting regular breathing and potential for translation. A “silver bullet” is unlikely to exist but a promising symptomatic strategy currently focuses on regulation of serotonergic function using highly selective serotonin type 1A agonists (5-HT_1A_). Here we address this newly emerging therapy for respiratory brainstem dysfunction in Rett syndrome and challenges for translation with a holistic perspective.

## Serotonin and autonomic control in rett syndrome

Deficiencies of 5-HT neurotransmission have been found both in humans suffering from Rett syndrome and in mouse models of the disease. In women with known MeCP2 mutations who met the clinical criteria for Rett syndrome, low spinal fluid levels of a 5-HT metabolite were found (Samaco et al., 2009). Low levels of 5-HT were also found in the brain of MeCP2 knockout male mice and were progressive with development (Ide et al., 2005; Viemari et al., 2005). But brain concentrations of tryptophan, the serotonin precursor, were comparable to wild-type control mice suggesting a failure of biosynthesis of serotonin (Ide et al., 2005).

Serotonin is an important regulatory neurotransmitter in the respiratory network. Generally it has a modulatory effect on breathing, increasing or decreasing post-synaptic excitability depending on the types of receptors expressed. Overall, the effect of 5-HT results in net stimulation of ventilation, and it is a significant component of ventilatory responses to CO_2_ (Richerson, 2004). Global deletion or acute inhibition of serotoninergic neurons in mice results in blunted respiratory responses to CO_2_ challenges (Hodges et al., 2008; Ray et al., 2011). Deletion of MeCP2 in male mice reduced CO_2_ sensitivity (Zhang et al., 2011) and selective blockade of serotonin re-uptake with citalopram corrected it (Toward et al., 2013). Recently, female mice with two different MeCP2 mutations have shown depressed CO_2_ chemosensitivity (Bissonnette et al., 2014). Imbalance in CO_2_ homeostasis was also found in human patients (Smeets et al., 2006). Forceful breathing and hyperventilation are a common occurrence in between episodes of apnea, and often result in hypocapnia (Smeets et al., 2006; Halbach et al., 2012). This combined with an upwards-shifted apneic threshold (Toward et al., 2013) could contribute to episodes of central apnea and oxygen desaturation (Southall et al., 1988). Hypocapnia and low oxygen can be powerful triggers of seizures or epilepsy-like episodes in Rett syndrome. In one patient, described as a forceful breather with epochs of Valsalva maneuvers, such episodes were successfully managed by administration of CO_2_ to correct hypocapnia (Smeets et al., 2006).

In addition to CO_2_ homeostasis, serotonin is also thought to promote regular breathing via activation of 5-HT_1A_ receptors in key brainstem sites involved in termination of inspiration (Richter et al., 2003). A 5-HT_1A_ partial agonist, buspirone, has been used to treat apneusis caused by surgical resection of a pontine astrocytoma in human (Wilken et al., 1997) and abnormal breathing in Rett syndrome (Andaku et al., 2005; Gokben et al., 2012). Systemic administration of a 5-HT_1A/7_ agonist, (R)-(+)-8-hydroxy-2-(di-n-propylamino) tetralin hydrobromide (+8-OH-DPAT) has also abolished spontaneous central apneas both in wild-type mice (Stettner et al., 2008) and MeCP2 deficient female mice (Abdala et al., 2010). More recently, systemic administration of 5-HT_1A_ agonists produced some of the most robust rescue of respiratory phenotype yet observed in multiple mouse models of Rett syndrome (Levitt et al., 2013; Abdala et al., in press).

**Figure 1:**
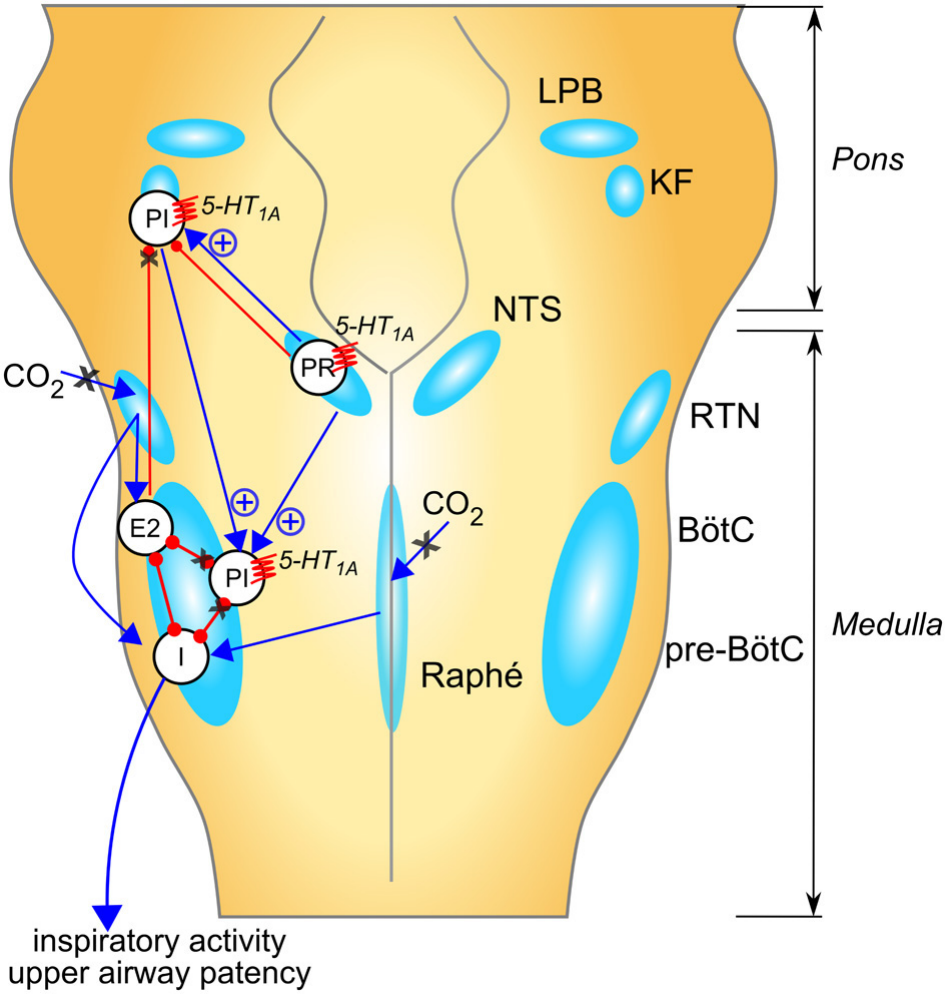
**Suggested mechanisms for respiratory rhythm disease in Rett syndrome and network targets of 5-HT_1A_ agonists**. Populations of respiratory neurons are shown in white circles (see below). Blue arrows indicate excitatory drive; red connectors with circle-ends indicate inhibitory drive. A healthy respiratory rhythm and pattern are critically dependent on the balance between excitatory and inhibitory synaptic drives to the “core” of mutually inhibitory respiratory neurons located in the BötC and pre-BötC (Smith et al., 2013). The disturbed rhythm in Rett syndrome seems to arise from an imbalance of drives to this core circuitry (indicated by black “X” when reduced, or blue “+” when enhanced); many mechanisms contribute to this: (i) weakened excitatory synaptic drives to and within the inspiratory “kernel” (Viemari et al., 2005), (ii) reduced CO_2_ sensitivity (Zhang et al., 2011; Toward et al., 2013; Bissonnette et al., 2014); (iii) excess descending post-inspiratory drive from the pontine parabrachial complex (Stettner et al., 2007; Voituron et al., 2010; Dhingra et al., 2013); which could be a consequence of loss of inhibitory drives to this area, including KF (Stettner et al., 2007; Abdala et al., 2010). In combination, these mechanisms would lead to disinhibition of PI populations, disruption of timing for termination of inspiration and expiratory length irregularity. Studies in humans and mice suggest that breath-holds and Valsalva maneuvers may be linked to active closure of the glottis implicating a failure in the ponto-medullary gating of central post-inspiratory activity, for a review; see Ramirez et al. (2013). 5-HT_1A_ receptors suppress specific inhibitory glycinergic neuron populations in the “core” of mutually inhibitory neurons with consequent disinhibition of inspiratory populations (Shevtsova et al., 2011). In addition, 5-HT_1A_ receptors can directly reduce the activity of neuron populations contributing to the descending post-inspiratory drive from the pons. I, inspiratory neuron population; PI, post-inspiratory neuron population; E2, late expiratory neuron population; PR, pulmonary stretch relay; LBP, lateral parabrachial nu; KF, Kölliker-Fuse nu; NTS, nucleus of the solitary tract; RTN, retrotrapezoid nu; BötC, Bötzinger complex; pre-BötC, pre-Bötzinger complex.

Figure 1 summarizes multiple suggested mechanisms for generation of breathing irregularity in Rett syndrome observed in mouse models and the potential ponto-medullary targets for 5-HT_1A_ agonists. It is noteworthy that 5-HT receptors are expressed in diverse brainstem regions and can mediate additive or opposing effects on respiratory control. For instance, activation of 5-HT_1A_ somatodendritic autoreceptors on raphe serotoninergic neurons reduced CO_2_ sensitivity (Corcoran et al., 2013) a potentially undesirable effect in RTT. However, a selective agonist of 5-HT_1A_ post-synaptic heteroreceptors effectively corrected the respiratory phenotype in a mouse model of RTT (Levitt et al., 2013). This suggests that the beneficial effects of 5-HT_1A_agonists on breathing are chiefly mediated by heteroreceptors.

Serotonin is also important for cardiac sympatho-vagal balance. Serotoninergic agonists shift the equilibrium between cardiac sympathetic and vagal drives toward the latter, which has a protective effect against ventricular fibrillation (Lehnert et al., 1987; McCall et al., 1994; Chen et al., 2008). Chen et al. (2008) showed that a 5-HT_1A/7_ agonist disinhibited vagal motor neurons via reduction of inputs from inhibitory neurons. In mice, deletion of 5-HT_1A_ receptors increased the susceptibility to sudden cardiac death under chronic stress (Carnevali et al., 2012). Interestingly, girls with Rett syndrome present reduced heart rate variability, as consequence of a reduced vagal tonus, which correlated with lower plasma levels of serotonin (Guideri et al., 1999, 2004). The reduced vagal tonus is progressive with development and clinical stage, and may explain the high risk of sudden death in Rett syndrome (Kerr et al., 1997; Guideri et al., 2001). In a cohort of patients with normal cardiac vagal drive, the increase of vagal tonus induced by selective 5-HT_1A_ agonists would be an undesirable side effect, placing them at risk of atrial arrhythmias. In patients with Rett syndrome, who display a shifted set point of cardiac parasympathetic drive, the increase of vagal tonus induced by selective 5-HT_1A_ agonists would be a particularly desirable effect.

## Sleep disordered breathing in rett syndrome

Another likely contributor for sympathetic overactivity in Rett syndrome is sleep disordered breathing. Recent polysomnography recordings in girls with Rett syndrome revealed that obstructive sleep apneas (OSAs) are highly prevalent in this population. During sleep, central apneas are less frequent and shorter when compared to wakefulness (Weese-Mayer et al., 2008). Even though central apneas are 10–30% more frequent than OSAs during sleep, the latter are clinically relevant in approximately 50% of the patients investigated, causing life threatening oxygen desaturations (SpO_2_ < 75%) (Hagebeuk et al., 2012; Carotenuto et al., 2013). In children, severe OSA has clear health implications, the common symptoms are: frequent awakenings, night terrors, poor concentration, irritability, behavioral problems, failure to thrive, enuresis, attention-deficit disorder, cardiopulmonary disease, and excessive daytime sleepiness (Waters et al., 2013). Most of these symptoms are easily confounded with the symptomatology of Rett syndrome itself. Unfortunately, this means that often, from a clinician's point of view, OSA diagnosis and treatment is not a priority in Rett patients.

In fact, the high prevalence of OSAs is relevant for the treatment of respiratory disturbances in Rett syndrome since they have potential to generate a reciprocal feedback loop with central apneas resulting in respiratory irregularities. It is well established that OSAs increase peripheral chemoreceptor sensitivity in humans independently of other underlying conditions (Narkiewicz et al., 1999). In turn, excess activation of the peripheral chemoreceptor reflex during apnea-induced desaturations in Rett syndrome, could aggravate hyperventilation episodes in between apneas and generate hypocapnia. This type of respiratory overshoot is particularly undesirable during sleep, as Pa_CO2_ reaches the apneic threshold resulting in periodic breathing (Dempsey et al., 2014). An overactive chemoreflex could also account for the sympatho-vagal imbalance common in patients with Rett (Guideri et al., 1999).

A pharmacotherapy capable of addressing all legs of this feedback loop would stand better chances of success in clinical practice. It is thought that, in the supine position, recruitment of the genioglossus can open a collapsed airway before arousal (Younes et al., 2012). For that reason, mechanisms that facilitate recruitment of hypoglossal motor neurons are an attractive target for pharmacotherapy. Rodent models revealed that the respiratory component of genioglossal activity is increased by 5-HT_1A_ receptor activation, a potentially beneficial effect (Besnard et al., 2007). Another potentially advantageous effect of this class of drugs is the reduction of epochs of REM sleep (Monti and Monti, 2000), since OSAs mostly occur in this phase. The lack of success in previous clinical trials (Kohler et al., 2009) using various older serotonin agonists may be due to their lack of receptor selectivity, weak partial agonism and/or poor pharmacokinetics (Fiorino et al., in press).

## Serotonin and mood control in rett syndrome

Particularly in Rett syndrome, mood disorder should not be viewed as a separate entity to autonomic dysfunction, as anxiety features could have a role in episodes of hyperventilation. Southall et al. (1988) observed that hyperventilation only occurred in wakefulness and was not necessarily preceded by hypoxemia, which suggests a central origin. Prolonged absences of inspiratory effort always followed hyperventilation. These girls also seemed agitated with increased muscle tonus and elevated heart rate, suggesting that anxiety may underlie these symptoms. SSRIs such as citalopram or fluoxetine are commonly used to treat anxiety disorders in a clinical setting. By preventing reuptake of 5-HT, these drugs modify serotonin levels and, indirectly, activation of serotonin receptors. It should also be noted that 5-HT receptors are expressed in diverse brain regions and can mediate complementary or sometimes opposing influence on mood and cognition (Newman-Tancredi, 2011). For example, activation of 5-HT_1A_ autoreceptors in the raphe nuclei can totally suppress electrical activity of serotonergic neurons and thus powerfully inhibit serotonin release in neuronal projection areas (Celada et al., 2013). In contrast, activation of post-synaptic cortical 5-HT_1A_ hetero-receptors expressed on glutamatergic pyramidal cells and/or GABAergic interneurons, elicits increased dopamine release in cortex (Santana et al., 2004; Bortolozzi et al., 2010), an effect which is associated with beneficial effects on mood deficits as well as positive effects on cognitive function (Phillips et al., 2004).

In the case of RTT patients, lower basal levels of 5-HT are reported (see above) and some of the mood deficits observed in Rett syndrome patients may therefore arise from deficiencies in serotonergic transmission (particularly via 5-HT_1A_ receptors) in specific brain regions. Such a hypothesis is reinforced by transgenic mouse studies that have provided insight into the developmental role of 5-HT_1A_ receptors (Akimova et al., 2009; Garcia-Garcia et al., 2014). Thus, mice with a genetically-elicited deletion of 5-HT_1A_ receptors (5-HT_1A_ KO mice) exhibit heightened anxiety-like behavior, as do mice with a heterozygote 5-HT_1A_ genotype that express about half of normal levels of 5-HT_1A_ receptor density. This suggests that even partial decreases in 5-HT_1A_ receptor expression (which may mimic the impaired serotonergic transmission observed in Rett syndrome) can elicit phenotypically-meaningful increases in anxiety levels. In contrast, when mice were engineered to overexpress 5-HT_1A_ receptors (Kusserow et al., 2004), they exhibited the opposite phenotype, with decreased anxiety-like behavior and increased hippocampal and striatal levels of serotonin. An additional study investigated the reversal of the anxiety-like phenotype of 5-HT_1A_ KO mice. In these mice, 5-HT_1A_ receptors were overexpressed in pyramidal neurons (but not GABAergic interneurons; Gross et al., 2002), in various forebrain regions, and this re-established normal behavioral responses. This study is significant because it shows that the “anxious” phenotype of the KO mice can be rescued, if 5-HT_1A_ receptor signaling is reintroduced to the relevant brain regions. Such observations support the assertion that treatments that augment 5-HT_1A_ receptor signaling in appropriate forebrain structures can attenuate anxiety symptoms.

The possibility that mood deficits seen in Rett syndrome patients arise from neurodevelopmental disruption of 5-HT_1A_ receptor expression is reinforced by studies showing that undisturbed expression of the 5-HT_1A_receptor is required in the 2nd and 3rd week of life for the emergence of a normal anxiety-like phenotype. Indeed, genetic disruption of 5-HT_1A_ receptors in mice at this time period elicits development of pathological levels of anxiety (Gross et al., 2002; Leonardo and Hen, 2008), an observation that can be mimicked by pharmacological blockade of 5-HT_1A_ receptors (Lo Iacono and Gross, 2008).

Taken together, the above considerations suggest that activation of 5-HT_1A_ receptors by administration of agonist drugs to Rett patients could attenuate anxiety symptoms in adults. In addition, if the heightened anxiety symptoms are the result of insufficient serotonergic activation during development, it may be speculated that treatment with a 5-HT_1A_ agonist at a young age may exert some measure of protection against the emergence of later anxiety symptoms. Nevertheless, considerable additional investigation is necessary to substantiate such hypotheses, notably because none of the above studies on transgenic 5-HT_1A_ mice was carried out in female MeCP2^+/−^ mice modeling Rett syndrome.

Indeed, studies of anxiety-like behavior in MeCP2^+/−^ mice have yielded somewhat divergent data. One study found that MeCP2^+/−^ female mice exhibited lower anxiety-like behavior in two different tests (Samaco et al., 2013). In contrast, another study in male mice expressing a truncated allele of MeCP2 (MeCP2^108/y^) (McGill et al., 2006) found that MeCP2^+/−^ mice show responses that are typical of increased anxiety in the same tests. The relationship between these observations and the heightened anxiety observed in individuals with Rett syndrome is unclear, but there is a clear need for additional investigation of anxiety-like behavior in RTT mice, particularly in response to clinically-employed anxiolytic drugs and 5-HT_1A_ receptor agonists.

## Dopaminergic interactions of serotoninergic agonists: implications for motor function in rett syndrome

In women with known MeCP2 mutations who met the clinical criteria for Rett syndrome, low spinal fluid levels of a dopamine metabolite were found (Samaco et al., 2009). Assuming that this is a marker for central dopamine levels, it may provide a neurochemical substrate for the devastating disturbances in motor function observed in Rett syndrome, characterized by loss of purposeful use of the hands, ataxia, tremors, gait apraxia, rigidity, and dystonia. Another indicator of compromised dopaminergic transmission is that, later in life, the women with Rett develop Parkinsonian features (Fitzgerald et al., 1990; Roze et al., 2007).

The precise role of serotonin in Rett syndrome motor deficits in currently unclear but some analogies may be drawn from the use of serotonergic agonists in the treatment of Parkinson's disease (PD)—which is also characterized by dopaminergic deficiencies leading to motor impairment. For example (i) 5-HT_1A_ receptor agonists reverse catalepsy induced by blockade of dopamine receptors with neuroleptics (McMillen et al., 1988; Wadenberg, 1996); (ii) (+)-8-OH-DPAT and sarizotan reduced L-DOPA-induced dyskinesia in monkeys with lesioned dopaminergic neurons in the substantia nigra (Iravani et al., 2006; Gregoire et al., 2009; Marin et al., 2009); and (iii) in clinical trials, sarizotan, buspirone, and tandospirone alleviated dyskinesia in PD patients (Bonifati et al., 1994; Kannari et al., 2002). At a neurochemical level, 5-HT_1A_ receptor agonists increase dopamine release in frontal cortex, as mentioned above, and modify the activity of cortico-striatal glutamatergic projections. Such influence, possibly involving inhibition of glutamate in the striatum may underlie the capacity of 5-HT_1A_ receptor agonists to facilitate movement control (Dupre et al., 2011; Huot et al., 2011). Table 1 lists drugs currently under clinical testing that possess serotonin 5-HT_1A_ receptor agonist activity.

**Table 1:** **Drugs currently under clinical testing that possess serotonin 5-HT_1A_ receptor agonist activity**.

**Drug**	**Clinical indication**	**Trade name®** or highest development	**Company**	**Mechanism of action**	**References**
Buspirone	Anxiety (GAD)	Buspar®	BMS	5-HT_1A_ partial agonist, D_2_ antagonist	Akimova et al., 2009
Tandospirone	Anxiety (GAD)	Sediel®	Dainippon Sumitomo	5-HT_1A_ partial agonist, D_2_ antagonist	Meltzer and Sumiyoshi, 2008
Vilazodone	Depression	Viibryd®	Clinical Data	SRI, 5-HT_1A_ partial agonist	Dawson and Watson, 2009
Vortioxetine	Depression	Brintellix®	Lundbeck/ Takeda	SRI; 5-HT_1A_ and 5-HT_1B_partial agonist; 5-HT_1D_, 5-HT_3_ and 5-HT_7_ antagonist	Mork et al., 2009
Flibanserin	Female hypoactive sexual desire disorder	Phase III	Sprout	5-HT_1A_ agonist, 5-HT_2A_ antagonist, D_4_ partial agonist	Stahl et al., 2011
Sarizotan	L- DOPA-induced dyskinesia in Parkinson's disease^a^	Phase III	Newron	5-HT_1A_ agonist, D_2_ partial agonist	Bartoszyk et al., 2004
Befiradol	L- DOPA-induced dyskinesia in Parkinson's disease	Phase II	Neurolixis	Selective 5-HT_1A_ full agonist	Colpaert, 2006
Eltoprazine	L- DOPA-induced dyskinesia in Parkinson's disease	Phase II	Amarantus	5-HT_1A_ and 5-HT_1B_partial agonist, 5-HT_2A_, 5-HT_2B_, 5-HT_2C_	Bezard et al., 2013
NLX-101 (F15599)	Rett syndrome	Phase I	Neurolixis	Selective post-synaptic 5-HT_1A_ “biased agonist”	Levitt et al., 2013

GAD, Generalized Anxiety Disorder; SRI, Serotonin Reuptake Inhibitor.

a Sarizotan may also be developed for Rett syndrome (http://www.newron.com/eng/Default.aspx?SEZ=3&PAG=141).

Insofar as such observations may be relevant to Rett syndrome, they suggest that activation of 5-HT_1A_ receptors may alleviate some of the disturbed motor function, including dyskinesia and dystonia, which constitute a source of poor movement control in RTT patients. Nevertheless, experimental investigation is necessary to substantiate this hypothesis, for example by examining the motor sensitivity of MeCP2 mice to dopamine receptor blockade (catalepsy induction) and whether 5-HT_1A_ receptor agonists can protect against such responses. It should also be noted that the therapeutic potential of current 5-HT_1A_ agonists suffers from some important limitations. Prominent among these is poor selectivity of the drugs with respect to cross-reacting targets. Indeed, buspirone also acts as a dopamine D2 receptor antagonist (Peroutka, 1985), and sarizotan is a weak D2 receptor partial agonist (Bartoszyk et al., 2004; Newman-Tancredi et al., 2005; Bruins Slot et al., 2006), and may act as a functional antagonist. This suggests again that high selectivity for 5-HT_1A_ receptors is an important requirement to avoid concurrent off-target blockade of dopamine receptors. Secondly, drugs such as eltoprazine, buspirone, and tandospirone possess only modest agonist efficacy at 5-HT_1A_ receptors. Thus, even at high doses, they are only able to partially activate the receptor (Newman-Tancredi et al., 2003), suggesting that optimal motor control by 5-HT_1A_ receptors may necessitate high-efficacy agonist stimulation. The novel 5-HT_1A_ receptor agonist, NLX-101 (also known as F15599) constitutes a promising advance in view of its exceptional 5-HT_1A_ receptor selectivity, high agonist efficacy and “biased agonist” profile (Newman-Tancredi et al., 2009), potently activating post-synaptic cortical 5-HT_1A_ heteroreceptors (Llado-Pelfort et al., 2010). *In vivo*, NLX-101 exhibits pro-motor influence in rats with dopaminergic lesions, potent antidepressant-like properties (Assie et al., 2010), pro-cognitive activity (Depoortere et al., 2010); and, in MeCP2 mice, reverses respiratory disturbance (Levitt et al., 2013), properties that would be desirable in a pharmacotherapy strategy for Rett syndrome.

## Conclusions

The severe autonomic phenotype of Rett syndrome has the greatest impact on patient's health and quality of life, closely followed by motor deficits. 5-HT_1A_ agonists have shown promising ability to alleviate brainstem, extrapyramidal and mood dysfunction in pre-clinical studies. In particular, a new class of selective “biased agonists” that target post-synaptic heteroreceptors shows less potential for competing or undesirable side effects. However, further rigorous pre-clinical testing in different mouse models of Rett syndrome is needed, especially in regards to motor dysfunction. In mouse models, the syndrome is reversible if MeCP2 function is rescued, but a cure applicable to humans is uncertain and may be many years away. In the meantime, there is need for drug therapies that are tolerated upon long term treatment and capable of alleviating the life-threatening autonomic symptoms.

## Author contributions

Ana P. Abdala and Adrian Newman-Tancredi drafted the manuscript; Ana P. Abdala, John M. Bissonnette, and Adrian Newman-Tancredi revised it critically for important intellectual content; and approved the final version to be published; and are accountable for all aspects of the work.

### Conflict of interest statement

Adrian Newman-Tancredi has received consulting and/or speaker honoraria from pharmaceutical companies. He is Chief Scientific Officer and stockholder at Neurolixis.
